# Adjuvant treatment strategy evolution and risk stratification for hormone receptor-positive, human epidermal growth factor receptor-2 negative early breast cancer in China

**DOI:** 10.1093/oncolo/oyae095

**Published:** 2024-05-23

**Authors:** Ying Fan, Danyang Ji, Mingxia Jiang, Yujing Tan, Yang Yang, Tianyi Li, Xiao Ma, Binghe Xu

**Affiliations:** Department of Medical Oncology, National Cancer Center/National Clinical Research Center for Cancer/Cancer Hospital, Chinese Academy of Medical Sciences and Peking Union Medical College, Beijing, People’s Republic of China; Department of Medical Oncology, National Cancer Center/National Clinical Research Center for Cancer/Cancer Hospital, Chinese Academy of Medical Sciences and Peking Union Medical College, Beijing, People’s Republic of China; Department of Medical Oncology, National Cancer Center/National Clinical Research Center for Cancer/Cancer Hospital, Chinese Academy of Medical Sciences and Peking Union Medical College, Beijing, People’s Republic of China; Department of Medical Oncology, National Cancer Center/National Clinical Research Center for Cancer/Cancer Hospital, Chinese Academy of Medical Sciences and Peking Union Medical College, Beijing, People’s Republic of China; Eli Lilly and Company, Shanghai, People’s Republic of China; Eli Lilly and Company, Shanghai, People’s Republic of China; Eli Lilly and Company, Shanghai, People’s Republic of China; Department of Medical Oncology, National Cancer Center/National Clinical Research Center for Cancer/Cancer Hospital, Chinese Academy of Medical Sciences and Peking Union Medical College, Beijing, People’s Republic of China

**Keywords:** breast cancer, HR+, HER2−, adjuvant therapy, recurrence, risk factors

## Abstract

**Background:**

Patients with hormone receptor-positive (HR+), human epidermal growth factor receptor-2 negative (HER2−) early breast cancer (EBC) with high-risk clinicopathological features face an increased risk of recurrence. This study explored the evolving treatment landscape and clinical outcomes in patients with EBC using a nationwide database.

**Patients and Methods:**

The study cohort comprised HR+/HER2−, stages 1-3, patients with EBC who underwent surgery and received adjuvant endocrine therapy (AET) from January 2013 to March 2021. High-risk patients were defined by ≥4 positive axillary lymph nodes, or 1-3 positive lymph node(s) with at least one high-risk feature (histologic grade 3, tumor size ≥5 cm, or Ki-67 ≥20%). A low-risk cohort included patients not meeting the criteria. Survival analysis was conducted with a cutoff of September 2021.

**Results:**

The study included 4088 eligible patients (1310 high-risk patients and 2778 low-risk patients). High-risk patients were more likely to receive adjuvant chemotherapy and radiotherapy compared to low-risk patients. From 2013 to 2021, an increasing proportion of patients received aromatase inhibitors and ovarian function suppression as part of their AET. The 2-, 5-, and 7-year invasive disease-free survival for high-risk cohort were 90.67%, 75.26%, and 57.10%, respectively, these rates were notably higher for low-risk cohort at 97.14%, 89.85%, and 84.83%. High-risk patients demonstrated a higher risk of recurrence or death compared with low-risk patients (hazard ratio, 2.38; 95% CI, 1.82-3.12).

**Conclusion:**

In the setting of standard or even intensive AET, patients with EBC with high-risk features still present high recurrence risk, highlighting the urgent need for innovative adjuvant treatment strategies.

## Teaser

This study investigated shifts in the adjuvant treatment pattern for HR+/HER2− early breast cancer in China over the past decade, and further explored the risk stratification in Chinese patients using data from a large-scale nationwide database.

**Implications for Practice**

Over the past decade in China, “high-risk” patients with early breast cancer, characterized by ≥4 positive axillary lymph nodes or 1-3 positive lymph node(s) with at least one high-risk feature (histologic grade 3, tumor size ≥5 cm, or Ki-67 ≥20%), received standard or intensive adjuvant therapy, particularly chemotherapy, radiotherapy, and aromatase inhibitors in combination with ovarian function suppression as part of their adjuvant endocrine therapy (ET). However, these “high-risk” patients still face a notably elevated risk of recurrence or death. This underscores the imperative for considering novel adjuvant treatment strategies, such as CDK4/6i in combination with ET, to reduce their risk of recurrence.

## Introduction

Breast cancer remains the most frequently diagnosed cancer among women, constituting a leading cause of cancer-related mortality.^1^ The most prevalent subtype, hormone receptor-positive (HR+), human epidermal growth factor receptor-2 negative (HER2−) breast cancer, constitutes approximately 70% of all breast cancer cases, irrespective of race, ethnicity, age, or clinical and pathological characteristics.^2-4^ Notably, the proportion of patients diagnosed at stages 1-3, corresponding to early breast cancer (EBC), is 92.3% in overall Chinese patients with breast cancer.^5^ Likewise, over 90% of patients with HR+/HER2− breast cancer are diagnosed at stages 1-3.^6^

The standard integrative treatment approach for HR+/HER2− EBC entails surgery, radiotherapy, chemotherapy, and adjuvant endocrine therapy (AET).^7,8^ Overall, patients diagnosed with this biological subtype of EBC exhibit promising prognoses relative to other molecular subtypes.^2,9^ However, a significant proportion, estimated at 20%-30%, still experience local or distal recurrence.^10-12^ Identifying individuals at high risk of recurrence, however, remains an ongoing debate, hampering the precision of personalized treatment strategies. Various studies have investigated individual risk factors associated with recurrence in patients with HR+/HER2− EBC, such as significant tumor size, extensive involvement of axillary lymph nodes (LNs), high histologic grade, and elevated Ki-67 expression.^13,14^ Previous clinical investigations based on Western populations defined a high-risk recurrence group by considering the number of positive LNs involved, coupled with other risk factors like tumor size, histologic grade, or Ki-67 expression.^13,15-18^ Applying these criteria, a nationwide retrospective study in the United States found that the high-risk subgroup, constituting 13.8% of the HR+/HER2− EBC population, exhibited over a 3-fold increase in the risk of recurrence compared to the low-risk group.^19^

Of note, distinct disparities in disease characteristics, incidence, and mortality distinguish Chinese patients with HR+/HER2− EBC from their Western counterparts.^20^ Treatment approaches for these patients have undergone rapid evolution in the last decade.^21^ However, the lack of well-acknowledged risk assessment criteria for identifying high-risk patients within the Chinese population remains a significant challenge. In light of these gaps, the current study aims to investigate shifts in the adjuvant treatment pattern for HR+/HER2− EBC in China over the past decade, and further explore the risk stratification in Chinese patients using data from a large-scale nationwide database.

## Methods

### Data source

This study used electronic medical record (EMR) data from the National Cancer Center Oncology Database. In December 2018, the National Health Commission of China organized the construction of the “National Anti-Tumor Drug Surveillance System (NATDSS)” by the National Cancer Center, and established the database. As the largest oncology database in China, it has covered medical records from major cancer or general hospitals across 27 provinces, compiling an expansive repository of information on over 10 million cancer patients diagnosed between 2013 and 2021. This extensive database encompasses both structured and unstructured medical information. The database maintains an annual linkage with the national Vital Registry, ensuring the accurate collection of death information. Among all the participating hospitals, 50 hospitals stand out for their real-time, automated upload of all EMR data to the NATDSS, as well as effective and rigorous quality control measures. Thus, the data of this study were exclusively drawn from these 50 participating hospitals, which comprise both cancer and general medical hospitals, spanning across 27 provinces in China. This diverse inclusion ensured a well-rounded representation of the Chinese patients with EBC. During the study period, a total of 221 846 adult female patients diagnosed with breast cancer were identified. Subsequently, 84 032 patients with EBC (specifically, stages 1-3) were included for further screening, excluding those with metastatic disease and those lacking staging information.

The study’s methodology adhered strictly to the ethical standards established by the Declaration of Helsinki, the Good Pharmacoepidemiology Practices. A central ethical review board provided Institutional Review Board approval (number: 22/198-3400) for the overall research protocol, which included a waiver for informed consent. All data used for research were thoroughly de-identified and included provisions to prevent re-identification, ensuring the maintenance of patient confidentiality.

### Patient selection

The study encompassed patients with HR+/HER2−, stages 1-3, EBC who had undergone surgery and received AET. Given its well-recognized prognosis for high recurrence and poor survival, patients with early triple-negative breast cancer (TNBC; defined as HR−/HER2−) were also included as a comparative group.

The inclusion criteria were as follows: 1) female patients with EBC aged 18 years or older; 2) initial diagnosis of breast cancer with a TNM stage of 1A-3C; 3) at least 2 recorded visits between January 1, 2013 and September 30, 2021; 4) no evidence of distant metastasis at the time of the initial breast cancer diagnosis; 5) receipt of definitive primary breast tumor surgery and adjuvant therapy; 6) documented HR+/HER2− or HR−/HER2− test results prior to the commencement of adjuvant therapy; and 7) an index window between January 1, 2013 and March 31, 2021, to allow a minimum of 6 months of follow-up. For patients with HR+/HER2−, the index date was defined as the start date of AET. For patients with TNBC, the index date was the start date of adjuvant therapy.

Exclusion criteria comprised those diagnosed with inflammatory breast cancer or those with another primary cancer 12 months before or 3 months after the initial diagnosis of breast cancer. Patients were also excluded if they lacked key clinical data such as tumor stage, treatment regimen, or survival information.

### Data collection

Patient demographic and clinical data were extracted. This encompassed information such as age, diagnosis, menopausal status (pre- or postmenopausal), HR and HER2 status, Ki-67 level, tumor size, number of positive LNs, histologic grade, TNM stage, neoadjuvant therapy, adjuvant therapy (which included endocrine therapy [ET], chemotherapy, and targeted therapy), treatment response, disease recurrence, and mortality information. Any disease recurrence or death event was manually validated by physicians based on the review of medical records.

### Definition of high-risk and comparative cohorts

Based on the criteria established in the previous study,^18^ we identified and classified high-risk patients (cohort A) as follows:

Cohort A1: patients with ≥4 positive LNs.
Cohort A2: those with 1-3 positive LN(s) in conjunction with at least one of the following criteria: a histologic grade of 3; a tumor size ≥5 cm; or a Ki-67 level ≥20%.

Patients with HR+/HER2− EBC who did not satisfy these high-risk criteria were categorized as the low-risk group (cohort B). This encompassed patients with 1-3 positive LNs, a histologic grade <3, a tumor size <5 cm, and a Ki-67 level <20% (cohort B1), and patients with 0 positive LNs. Additionally, patients with early-stage TNBC were classified as cohort C.

### Statistical analysis

Baseline patient characteristics and treatment patterns were described using descriptive statistics. Continuous variables are presented as mean values with SD, whereas categorical variables are displayed as frequency and percentage (excluding the missing data in the denominator). Invasive disease-free survival (IDFS) was defined as the time from the initiation of AET for HR+/HER2− EBC or adjuvant therapy for TNBC to invasive ipsilateral breast tumor recurrence, local/regional invasive breast cancer recurrence, distant recurrence, invasive contralateral breast cancer, second primary invasive non-breast invasive cancer, or death from any cause, whichever occurred first. Distant relapse-free survival (DRFS) was defined as the time from the initiation of AET for HR+/HER2− EBC or adjuvant therapy for TNBC to the first distant recurrence or death from any cause. Overall survival (OS) was defined as the time from the initiation of AET for HR+/HER2− EBC or adjuvant therapy for TNBC to death from any cause. Survival curves for IDFS, DRFS, and OS were constructed using the Kaplan-Meier method. The 1-, 2-, 3-, 4-, 5-, 6-, and 7-year IDFS, DRFS, and OS rates were reported, each accompanied by their 95% CIs. Multivariable Cox proportional hazards regression models were used to estimate hazard ratios (HRs) and 95% CIs, adjusting for predefined risk factors. These factors were selected based on physicians’ clinical experience and included age, menopausal status, and (neo) adjuvant treatment patterns. In the case of missing data within the adjusted factors in the multivariable Cox regression analysis, patients with missing data were excluded, as the proportion of missing data was not extensive enough to warrant imputation. All statistical analysis was performed using SAS v9.4 software. A *P*-value of <.05 indicated statistical significance.

## Results

### Study cohorts and patient characteristics

**Figure 1.**
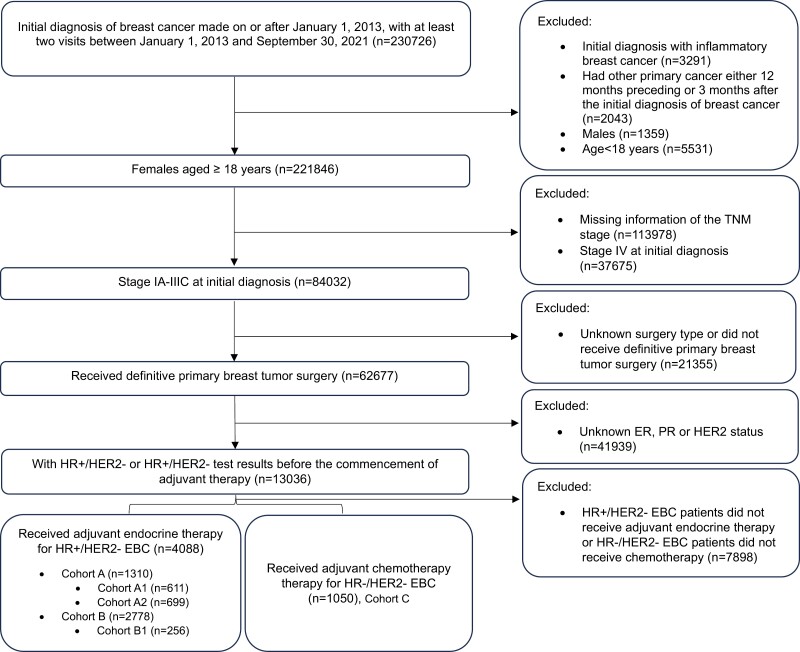
Patient flowchart. Cohort A: ≥4 positive LNs (cohort A1), or those with 1-3 positive LN(s) in conjunction with at least one of the following criteria: histologic grade of 3; tumor size ≥5 cm; or Ki-67 level ≥20% (cohort A2). Cohort B: 1-3 positive LN(s), histologic grade <3, tumor size <5 cm and Ki-67 level <20% (cohort B1), or node-negative patients. Cohort C: early-stage triple-negative breast cancer. Abbreviations: ER, estrogen receptor; PR, progesterone receptor; HER2, human epidermal growth factor receptor-2; HR, hormone receptor; EBC, early breast cancer.

A total of 230 726 patients diagnosed with breast cancer from January 2013 to September 2021 were initially screened for this study. After excluding ineligible patients and those with missing information on tumor stage, a cohort of 84 032 patients with EBC was initially identified. Subsequently, additional exclusions were made for patients lacking information on breast cancer surgery type, ER/PR, and HER2 status. This refined the cohort to 13 036 patients diagnosed with stages 1-3 EBC who underwent definitive primary breast cancer surgery. Further refinement involved applying specific inclusion criteria for adjuvant therapy, resulting in the identification of 4088 patients diagnosed with HR+/HER2− EBC for inclusion in the study. Cohort A consisted of 1310 (32.0%) patients and cohort B comprised 2778 (68.0%) patients. In cohort A, there were 611 patients further classified as cohort A1 and 699 patients as cohort A2. In cohort B, 256 patients were further classified as cohort B1. Besides, 1050 patients with TNBC were included in cohort C (Figure 1). The mean ages at initiation of adjuvant therapy were 51, 52, and 50 years for cohorts A, B, and C, respectively. Over 40% of patients in each cohort were premenopausal. The proportion of patients with stage 3 tumors was notably higher in cohort A compared to cohort B (43.1% vs 4.2%; Table 1).

**Table 1.** Baseline characteristics of patients with EBC.

Characteristic	Cohort A (*n* = 1310)	Cohort B (*n* = 2778)	Cohort C (*n* = 1050)
Age (years)	51 (11.1)	52 (10.7)	50 (10.1)
Premenopausal status			
Yes	453 (42.9)	953 (44.2)	350 (45.4)
No	604 (57.1)	1202 (55.8)	421 (54.6)
Progesterone receptor status			
Positive	1113 (88.5)	2449 (92.3)	0
Negative	144 (11.5)	204 (7.7)	1050 (100.0)
Estrogen receptor status			
Positive	1220 (98.3)	2586 (98.4)	0
Negative	21 (1.7)	41 (1.6)	1050 (100.0)
Ki-67 (%)			
<20	280 (23.0)	1186 (46.8)	79 (9.1)
≥20	938 (77.0)	1347 (53.2)	785 (90.9)
Number of positive lymph nodes			
0	0	2522 (90.8)	628 (61.6)
1-3	699 (53.4)	256 (9.2)	273 (26.8)
≥4	611 (46.6)	0	118 (11.6)
Histologic grade			
1	39 (4.0)	191 (9.6)	21 (2.8)
2	640 (64.8)	1455 (72.8)	270 (36.1)
3	308 (31.2)	353 (17.7)	457 (61.1)
Tumor size (cm)			
≥5	126 (12.0)	67 (2.9)	47 (5.7)
<5	922 (88.0)	2229 (97.1)	782 (94.3)
TNM stage			
1	46 (3.5)	1347 (48.5)	264 (25.1)
2	700 (53.4)	1314 (47.3)	633 (60.3)
3	564 (43.1)	117 (4.2)	153 (14.6)

Data are *n* (%) or mean (SD). Percentage is based on non-missing data.

Cohort A: ≥4 positive LNs, or those with 1-3 positive LN(s) in conjunction with at least one of the following criteria: histologic grade of 3; tumor size ≥5 cm; or Ki-67 level ≥20%. Cohort B: 1-3 positive LNs, histologic grade <3, tumor size <5 cm, and Ki-67 level <20%; or node-negative patients. Cohort C: early-stage triple-negative breast cancer.

### Treatment patterns of HR+/HER2− EBC

The proportions of patients receiving neoadjuvant therapy (27.8% vs 10.4%), adjuvant radiotherapy (72.1% vs 45.6%), and adjuvant chemotherapy (74.9% vs 63.7%) in cohort A were higher than those in cohort B, with taxane and anthracycline-containing regimen (sequential anthracyclines and taxanes [AC-T], concurrent anthracyclines and taxanes [TAC]) accounting for 63.4% versus 29.1%. Nearly half of the patients (49.9%) in cohort B received taxane-containing, anthracycline-free chemotherapy (TC). The overall proportions of patients with taxane-containing regimens were 63.7% in cohort A and 50.4% in cohort B. The primary adjuvant treatment regimen for patients with HR+/HER2− EBC was a combination of chemotherapy and ET (68.8% in cohort A vs 59.2% in cohort B). The main AET regimen for patients with HR+/HER2− EBC predominantly involved aromatase inhibitors (AI; 60.8% in cohort A vs 48.8% in cohort B), followed by selective estrogen receptor modulators (34.6% in cohort A vs 49.0% in cohort B; Table 2).

**Table 2.** Treatment patterns of HR+/HER2− EBC.

Treatment	Cohort A (*n* = 1310)	Cohort A1 (*n* = 611)	Cohort A2 (*n* = 699)	Cohort B (*n* = 2778)
Previous neoadjuvant therapy				
Yes	364 (27.8)	238 (39.0)	126 (18.0)	290 (10.4)
No	946 (72.2)	373 (61.0)	573 (82.0)	2488 (89.6)
Adjuvant radiotherapy				
Yes	944 (72.1)	487 (79.7)	457 (65.4)	1268 (45.6)
No	366 (27.9)	124 (20.3)	242 (34.6)	1510 (54.4)
Adjuvant treatment pattern				
Chemotherapy and endocrine therapy	901 (68.8)	400 (65.5)	501 (71.7)	1646 (59.2)
Endocrine therapy	327 (25.0)	164 (26.8)	163 (23.3)	1002 (36.1)
Targeted therapy^1^, chemotherapy, and endocrine therapy	80 (6.1)	45 (7.4)	35 (5.0)	124 (4.5)
Targeted therapy^1^ and endocrine therapy	2 (0.1)	2 (0.3)	0	6 (0.2)
Adjuvant chemotherapy				
Yes	981 (74.9)	445 (72.8)	536 (76.7)	1770 (63.7)
No	329 (25.1)	166 (27.2)	163 (23.3)	1008 (36.3)
Adjuvant chemotherapy regimen				
Taxane and anthracycline-containing (AC-T, TAC)	622 (63.4)	283 (63.6)	339 (63.2)	515 (29.1)
Taxane-containing, anthracycline-free (TC)	212 (21.6)	80 (18.0)	132 (24.6)	884 (49.9)
Anthracycline-containing, taxane-free (AC)	83 (8.5)	40 (9.0)	43 (8.0)	267 (15.1)
Others^2^	64 (6.5)	42 (9.4)	22 (4.1)	104 (5.9)
Adjuvant endocrine therapy				
Selective estrogen receptor modulator^3^	453 (34.6)	207 (33.9)	246 (35.2)	1361 (49.0)
Plus ovarian function suppression	136 (10.4)	64 (10.5)	72 (10.3)	239 (8.6)
Aromatase inhibitor	797 (60.8)	372 (60.9)	425 (60.8)	1357 (48.8)
Plus ovarian function suppression	219 (16.7)	108 (17.7)	111 (15.9)	162 (5.8)
Anastrozole	240 (18.3)	114 (18.7)	126 (18.0)	410 (14.8)
Letrozole	359 (27.4)	170 (27.8)	189 (27.0)	667 (24.0)
Exemestane	198 (15.1)	88 (14.4)	110 (15.7)	280 (10.1)
Others^4^	60 (4.6)	32 (5.2)	28 (4.0)	60 (2.2)

Data are *n* (%). For the categories in “adjuvant chemotherapy regimen,” percentages were calculated based on total number of patients who received adjuvant chemotherapy therapy in each specific cohort; for categories in “adjuvant endocrine therapy,” percentages were calculated based on total number of patients who received adjuvant endocrine therapy in each specific cohort.

A: anthracycline; C: cyclophosphamide; T: taxane.

Cohort A: ≥4 positive LNs (cohort A1), or those with 1-3 positive LN(s) in conjunction with at least one of the following criteria: histologic grade of 3; tumor size ≥5 cm; or Ki-67 level ≥20% (cohort A2). Cohort B: 1-3 positive LNs, histologic grade <3, tumor size <5 cm, and Ki-67 level <20%; or node-negative patients.

^1^Targeted therapy includes CDK4/6 inhibitors, PARP inhibitors and anti-angiogenic agents, etc.

^2^Other categories include cyclophosphamide, chloroquine, capecitabine and fluorouracil, etc.

^3^Selective estrogen receptor modulators include tamoxifen and toremifene.

^4^Other categories include: ovarian function suppression, ovarian function suppression plus fulvestrant, ovarian function suppression plus megestrol, and megestrol.

From 2013 to 2021, the proportion of patients with HR+/HER2− EBC receiving AIs as part of their AET increased from 36.4% to 58.2%, while the proportion of patients receiving selective estrogen receptor modulator (toremifene and tamoxifen) saw a decrease from 60.8% to 38.7%. The proportion of patients combining AI with ovarian function suppression (OFS) also demonstrated an increase over the study period from 2.2% to 12.1%. Regarding the chemotherapy, the proportions of taxane and anthracycline-containing regimen (AC-T, TAC) were 37.3%, 44.0%, 40.5% during 2013-2015, 2016-2018, and 2019-2021; taxane-containing, anthracycline-free regimen (TC) accounted for approximately 40% during the study periods; while anthracyclines-containing, taxane-free regimen (AC) saw a decrease from 17.5% during 2013-2015 to 11.5% during 2019-2021 (Table 3).

**Table 3.** Treatment patterns of HR+/HER2− EBC, 2013-2021.

Treatments	2013-2015 (*n* = 401)	2016-2018 (*n* = 1306)	2019-2021 (*n* = 2381)
Previous neoadjuvant therapy			
Yes	59 (14.7)	170 (13.0)	425 (17.8)
No	342 (85.3)	1136 (87.0)	1956 (82.2)
Adjuvant treatment pattern			
Chemotherapy and endocrine therapy	251 (62.6)	827 (63.3)	1469 (61.7)
Endocrine therapy	137 (34.2)	390 (29.9)	802 (33.7)
Targeted therapy^1^, chemotherapy, and endocrine therapy	12 (3.0)	87 (6.7)	105 (4.4)
Targeted therapy^1^ and endocrine therapy	1 (0.2)	2 (0.2)	5 (0.2)
Adjuvant endocrine therapy			
Selective estrogen receptor modulator^2^	244 (60.8)	649 (49.7)	921 (38.7)
Plus ovarian function suppression	48 (12.0)	112 (8.6)	215 (9.0)
Aromatase inhibitor	146 (36.4)	623 (47.7)	1385 (58.2)
Plus ovarian function suppression	9 (2.2)	85 (6.5)	287 (12.1)
Anastrozole	43 (10.7)	168 (12.9)	439 (18.4)
Letrozole	70 (17.5)	325 (24.9)	631 (26.5)
Exemestane	33 (8.2)	130 (10.0)	315 (13.2)
Others^3^	11 (2.7)	34 (2.6)	75 (3.1)
Adjuvant chemotherapy regimen			
Taxane and anthracycline-containing (AC-T, TAC)	98 (37.3)	402 (44.0)	637 (40.5)
Taxane-containing, anthracycline-free (TC)	104 (39.5)	365 (39.9)	627 (39.8)
Anthracycline-containing, taxane-free (AC)	46 (17.5)	123 (13.5)	181 (11.5)
Others^4^	15 (5.7)	24 (2.6)	129 (8.2)

Data are *n* (%). A: anthracycline; C: cyclophosphamide; T: taxane.

^1^Targeted therapy includes CDK4/6 inhibitors, PARP inhibitors and anti-angiogenic agents, etc.

^2^Selective estrogen receptor modulators include tamoxifen and toremifene.

^3^Other categories include ovarian function suppression, ovarian function suppression plus fulvestrant, ovarian function suppression plus megestrol, and megestrol.

^4^Other categories include cyclophosphamide, chloroquine, capecitabine and fluorouracil, etc.

### Clinical outcomes in high-risk and low-risk cohorts

**Figure 2.**
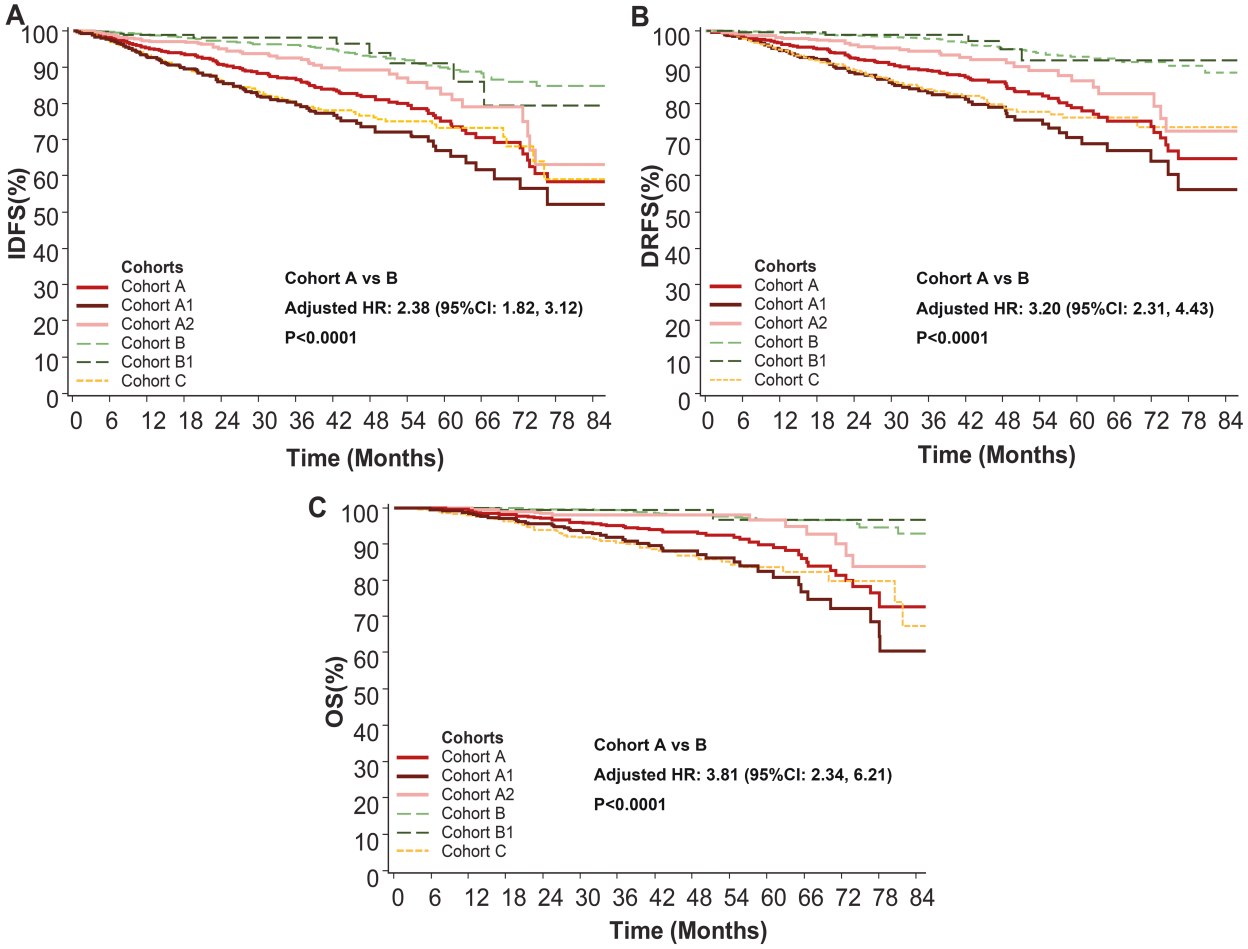
Kaplan-Meier curves for invasive disease-free survival (IDFS), distant relapse-free survival (DRFS), and overall survival (OS) of early breast cancer. Cohort A: ≥4 positive LNs (cohort A1), or those with 1-3 positive LN(s) in conjunction with at least one of the following criteria: histologic grade of 3; tumor size ≥5 cm; or Ki-67 level ≥20% (cohort A2). Cohort B: 1-3 positive LN(s), histologic grade <3, tumor size <5 cm and Ki-67 level <20% (cohort B1), or node-negative patients. Cohort C: early-stage triple-negative breast cancer. Hazard ratio (HR) and 95% CI were estimated from Cox proportional hazards regression models with adjustments for age, menopausal status, neoadjuvant therapy, adjuvant chemotherapy and radiotherapy. Two hundred and fifty-three patients in cohort A and 623 patients in cohort B were excluded from the analysis due to missing adjustment variables.

The 2-year IDFS rates for cohorts A, B, and C were 90.67%, 97.14%, and 86.50%, respectively. The 5-year IDFS rates were 75.26%, 89.85%, and 73.34% for the same cohorts; furthermore, 7-year IDFS rates were 57.10%, 84.83%, and 55.21%. The pattern observed in the DRFS and OS mirrored that of the IDFS. When comparing to patients in cohort A to those in cohort B, a higher risk of recurrence or death was observed (adjusted HR, 2.38; 95% CI, 1.82-3.12). Similarly, patients in cohort A had a higher risk of distant recurrence (adjusted HR for DRFS, 3.20; 95% CI, 2.31-4.43) and death (adjusted HR for OS, 3.81; 95% CI, 2.34-6.21; Figure 2).

### Clinical outcomes in high-risk subgroups

The IDFS, DRFS, and OS rates for both cohorts A1 and A2 were lower than cohort B (Figure 2). The adjusted HRs for cohort A1 and A2 in comparison to cohort B in IDFS were 3.44 (95% CI, 2.55-4.65) and 1.50 (95% CI, 1.05-2.14), respectively. Corresponding trends were observed in the HRs for DRFS and OS (Supplementary Table S1).

Upon further stratification by axillary LN involvement number, patients in cohort A2 with 1-3 LN involvement and any of these high-risk factors (grade 3, tumor size ≥5 cm, or Ki-67 level ≥20%) faced an elevated risk of recurrence compared to cohort B. This risk escalated for IDFS with the increasing number of involved LNs: 1 positive LN + high-risk factors (HR: 1.78; 95% CI: 1.17-2.68), 2 positive LNs + high-risk factors (HR: 1.88; 95% CI: 1.08-3.28), and 3 positive LNs + high-risk factors (HR: 2.43; 95% CI: 1.27-4.64). The trends observed for DRFS and OS rates followed those of IDFS (Supplementary Table S2).

## Discussion

This was the first study based on the nationwide oncology database in China, which represented a well-distributed, nationwide study population. This study provided important insights into the risk stratification and adjuvant treatment evolution in patients with HR+/HER2− EBC over the past decade. In this study, both short-term (2-year) and long-term (7-year) risks of recurrence and death among patients with EBC demonstrated that the “high-risk criteria” could serve as an effective tool for stratifying recurrence risk in Chinese patients with HR+/HER2− EBC.

Our study underscored the implication of the “high-risk criteria” in identifying the patients with HR+/HER2− EBC with poor prognosis. Even patients with a solitary positive LN and concurrent high-risk factors displayed a higher risk of recurrence in comparison to low-risk patients, suggesting the necessity for intensive treatment in these high-risk patients. A previous retrospective study in the United States using electronic health records and the same criteria for classifying high-risk patients with HR+/HER2− EBC reported an increased 5-year recurrence risk (29.8% high-risk vs. 9.1% low-risk; HR 3.07, 95% CI 2.45-3.83).^19^ Similar trends were observed for distant recurrence/death rates (HR 3.15, 95% CI 2.49-3.97). Our study, despite observing a younger Chinese population with fewer stage 3 patients and a higher proportion of high-risk patients, identified consistent trends in recurrence rates for high-risk versus non-high-risk groups. This suggests good generalizability of risk stratification across geographic regions.^19^ Moreover, a US study using SEER registry data (2010-2015) demonstrated a significantly elevated 5-year mortality risk among high-risk patients with HR+/HER2− EBC.^22^ Of note, we also included patients with TNBC, known for their unfavorable long-term prognosis,^6^ as a comparative group in our study. Comparing with TNBC, HR+/HER2− EBC was considered as a subtype with a better prognosis. Strikingly, our study observed that HR+/HER2− high-risk EBC cohort (cohort A) demonstrated a long-term recurrence risk (beyond 5 years) paralleling that of the TNBC cohort, hinting at similar recurrence dynamics across these diverse patient populations. This further enhances the potential necessity of risk stratification in patients with HR+/HER2− EBC, and intensive treatment should not be ignored in patients who meet the high-risk criteria.

Compared to the low-risk cohort, a notably higher proportion in high-risk patients with HR+/HER2− EBC receiving neoadjuvant therapy and adjuvant chemotherapy or radiotherapy was observed. Previous studies showed the benefit of incorporating a taxane in the adjuvant chemotherapy for patients with EBC, taxanes should be considered as the therapy backbone in standard cytotoxic treatment of EBC.^23,24^ Predominantly, high-risk patients opted for taxanes and anthracyclines-containing chemotherapy regimen in the present study. A recent meta-analysis suggested a superior efficacy of AIs over tamoxifen in premenopausal patients undergoing OFS.^25^ Over the past decade, we have discerned a shift in treatment patterns for HR+/HER2− EBC. AIs have become the principal choice for AET, with an increasing trend of combination with OFS. These changes indicate a shift in clinical decision-making, adapting to the specific disease characteristics of the Chinese population. These evolutions highlight a trend toward intensive treatment approaches. However, the present study revealed that despite high-risk patients receiving intensive adjuvant therapeutic strategy, comprising taxanes + anthracyclines-based chemotherapy, radiotherapy in combination with ET plus OFS, the prognosis remains unfavorable, and nearly 25% of high-risk patients experienced recurrence or death within 5 years of AET treatment. Thus, personalized treatment strategies are needed to enhance prognosis. Recently, two phase 3 trials demonstrated CDK4/6i in combination with ET showed a significant decrease in recurrence risk of HR+/HER2− EBC.^26,27^ At the pivotal 5-year milestone for monarchE study, abemaciclib benefit was sustained with an increase in absolute IDFS (Δ7.6%) and DRFS (Δ6.7%) benefit in the high-risk patients with EBC. Of note, the present study used the same “high-risk” criteria as the monarchE trial. Thus, considering the current intensive adjuvant chemo-/radiotherapy, the CDK4/6i should also be contemplated into standard ET strategy to further decrease the recurrence risk in Chinese high-risk patients.

We achieved a comprehensive data capture, encompassing diagnostic information such as tumor stage and pathological type, treatment details, and patient outcomes. The integration of real-time medical information systems across hospitals not only ensured the timely updates of data but also maintained a consistently high level of data quality. This database was annually linked to China Vital Registry to obtain death information, which enhance the reliability and accuracy of the long-term OS data. Despite these strengths, the study also bore some limitations. For instance, only patients with complete variable information were included, potentially introducing selection bias by excluding patients with incomplete medical records. The final analysis of 5138 patients with EBC who underwent definitive primary breast cancer surgery and received adjuvant treatment only accounted for approximately 6% of the initial 84 032 patients with EBC. It was essential to acknowledge that the final study population, albeit carefully selected to meet specific criteria, may pose limitations on the generalization of the study results. The exclusion of patients with incomplete medical records and the relatively small proportion of the total EBC cohort included in the analysis could impact the broader applicability of the findings to the entire population of interest. Additionally, despite the adjustment for certain potential confounding factors, we still cannot rule out the possibility of residual confounding in the results.

## Conclusion

In conclusion, this study found that Chinese patients with HR+/HER2− EBC meeting the high-risk criteria face a significantly elevated risk of recurrence and death, even when subjected to standard or intensive adjuvant therapy. This underscores the imperative for considering novel adjuvant treatment strategies, such as CDK4/6i in combination with ET, to reduce their risk of recurrence.

## Supplementary material

Supplementary material is available at *The Oncologist* online.

oyae095_suppl_Supplementary_Material

## Data Availability

The data underlying this article will be shared on reasonable request to the corresponding author.
