# Supplementary material for: Adjuvant treatment strategy evolution and risk stratification for hormone receptor-positive, human epidermal growth factor receptor-2 negative early breast cancer in China
Source: Oncologist. 2024 May 23;29(9):e1104–12. doi: 10.1093/oncolo/oyae095 (PMC11379641; doi:10.1093/oncolo/oyae095)
Supplement: oyae095_suppl_Supplementary_Material [file oyae095_suppl_supplementary_material.docx]

**Supplementary Materials**

**Supplementary Table 1**. Risk of recurrence and death in HR+, HER2- early breast cancer

| **Cohorts ^1^** | **Event/total ^2^** | **HR (95%CI) ^3^** | **P value** |
| --- | --- | --- | --- |
| **Invasive disease-free survival** |  |  |  |
| Cohort B | 100/2155 | Ref. |  |
| Cohort A | 134/1057 | 2.38 (1.82,3.12) | <.0001 |
| *Cohort A1* | 88/469 | 3.44 (2.55,4.65) |  |
| *Cohort A2* | 46/588 | 1.50 (1.05,2.14) |  |
| Cohort C | 126/771 | 4.62(3.49,6.11) |  |
| **Distant relapse-free survival** |  |  |  |
| Cohort B | 61/2155 | Ref. |  |
| Cohort A | 106/1057 | 3.20 (2.31,4.43) | <.0001 |
| *Cohort A1* | 73/469 | 4.86 (3.40,6.94) |  |
| *Cohort A2* | 33/588 | 1.82 (1.19,2.80) |  |
| Cohort C | 100/771 | 6.58(4.68,9.27) |  |
| **Overall survival** |  |  |  |
| Cohort B | 25/2155 | Ref. |  |
| Cohort A | 53/1057 | 3.81 (2.34,6.21) | <.0001 |
| *Cohort A1* | 40/469 | 6.47 (3.84,10.92) |  |
| *Cohort A2* | 13/588 | 1.68 (0.85,3.30) |  |
| Cohort C | 56/771 | 9.36(5.65,15.50) |  |

^1^ **Cohort A**: ≥ 4 positive LNs (**Cohort A1**), or those with 1-3 positive LN(s) in conjunction with at least one of the following criteria: histologic grade of 3; tumor size ≥5 cm; or Ki-67 level ≥20% (**Cohort A2**). **Cohort B**: 1-3 positive LNs, histologic grade <3, tumor size <5 cm, and Ki-67 level <20%; or node-negative patients. **Cohort C**: early-stage triple-negative breast cancer.

^2^ 253 patients in cohort A and 623 patients in cohort B were excluded from the analysis due to missing adjustment variables in Cox proportional hazards regression models.

^3^ HR and 95%CI were estimated from Cox proportional hazards regression models with adjustments for age, menopausal status, neoadjuvant therapy, adjuvant chemotherapy and radiotherapy.

CI, confidence interval; HR, hazard ratio

**Supplementary Table 2.** Relative risk of recurrence and death for early breast cancer patients in Cohort A2 vs Cohort B (reference group), stratified by number of lymph nodes + high-risk factors*

| Risk Groups | HR  _iDFS_（95% CI） | HR _DRFS_（95% CI） | HR _OS_（95% CI） |
| --- | --- | --- | --- |
| 1 positive LN + high-risk factors* | 1.78 (1.17,2.68) | 2.29 (1.40,3.74) | 1.54 (0.64,3.72) |
| 2 positive LNs + high-risk factors* | 1.88 (1.08,3.28) | 2.08 (1.04,4.17) | 2.17 (0.76,6.18) |
| 3 positive LNs + high-risk factors* | 2.43 (1.27,4.64) | 4.24 (2.18,8.24) | 3.87 (1.36,11.05) |

* High risk factors meet at least one of the following conditions: tumor size ≥5 cm, tumor grade of 3, Ki67 ≥ 20%. HR and 95%CI were estimated from Cox proportional hazards regression models without multiple-factor adjustments due to limited sample size in subgroups.

**Cohort B**: 1-3 positive LNs, histologic grade <3, tumor size <5 cm and Ki-67 level <20%, or node-negative patients.

IDFS, invasive disease-free survival; DRFS, distant relapse free survival; OS, overall survival; LN, lymph node; HR, hazard ratio; CI, confidence interval.
